# Year in Review 2025: Noteworthy Literature in Cardiac Anesthesiology

**DOI:** 10.1177/10892532261437742

**Published:** 2026-03-26

**Authors:** Aubrey Yao, David Asseff, Orode Badakhsh, Ala Jamal, David Li, Hong Liu, Gregory Peterfreund, Nathaen Weitzel

**Affiliations:** 1 Department of Anesthesiology and Pain Medicine, 8789 University of California, Davis Health, Sacramento, CA, USA

**Keywords:** cardiac anesthesia, acute kidney injury, coagulopathy management, nitric oxide, prothrombin complex concentrate, acute normovolemic hemodilution, acute renal failure, opioid-sparing, delirium

## Abstract

Cardiac anesthesiology continues to evolve as new evidence reshapes practice across multiple domains of patient care. The pace and volume of publication make it increasingly difficult for clinicians to identify studies that are most likely to change day-to-day practice. We performed a structured search of major bibliographic databases and targeted high-impact journals to identify significant cardiac anesthesia research published in 2025. Of 639 records identified, 24 publications were selected by three reviewers based on methodological rigor, clinical relevance, and anticipated impact on perioperative management. The included literature clustered into six themes: prevention of acute kidney injury, blood conservation and coagulation management, multimodal analgesia and opioid-sparing strategies, Enhanced Recovery After Surgery (ERAS) protocols with emphasis on ventilation strategies and delirium prevention, point-of-care ultrasound applications, and workforce sustainability. Together, these studies highlight a continued shift from reactive rescue to proactive optimization—before, during, and after cardiac surgery. This review summarizes the most practice-informing cardiac anesthesiology publications from 2025 to support evidence-based decision-making at the bedside.

## Introduction

Cardiac anesthesiology remains at the leading edge of perioperative innovation.^1^ Daily practice requires high-stakes decisions in hemodynamic management, organ protection, coagulation and transfusion strategy, and postoperative recovery—often in older patients with significant comorbidities undergoing complex procedures. At the same time, the medical literature has expanded to a scale that makes “keeping up” unrealistic. The challenge is no longer access to information, but rather deciding which new evidence is sufficiently rigorous and clinically relevant to warrant changes in practice. This annual series, initiated in *Seminars in Cardiothoracic and Vascular Anesthesia* in 2013^2^ was developed to meet that need by curating studies most likely to influence perioperative care. In this installment, we synthesize key cardiac anesthesiology literature published in 2025 using similar methods to previous years,^1^ focusing on publications with clear applicability to contemporary adult cardiac surgical practice.

Across the selected studies, several themes recur. First, kidney protection remains a hot topic, with multiple investigations evaluating strategies to reduce perioperative acute kidney injury in high-risk populations. Second, blood conservation and coagulation management continue to evolve with renewed attention to techniques such as acute normovolemic hemodilution (ANH) and more targeted replacement of coagulation factors. Third, multimodal analgesia and opioid-sparing pathways are increasingly being used in cardiac Enhanced Recovery After Cardiac Surgery (ERACS) programs, along with efforts to optimize ventilation and reduce postoperative delirium. Fourth, point-of-care ultrasound continues to expand beyond procedural guidance into broader hemodynamic assessment and perioperative decision-making. Finally, the literature reflects growing recognition that workforce sustainability, burnout, staffing models, and training paradigms are inseparable from safe and high-quality care.

The intersection of these topics underscores a broader shift in cardiac anesthesiology: from reactive intervention to proactive optimization. Whether through preoperative blood conservation planning, intraoperative renal protection strategies, or structured ERAS pathways that begin before incision and extend through hospital discharge, contemporary practice increasingly emphasizes prevention and early intervention. This review presents 24 studies selected for their methodological rigor, clinical relevance, and capacity to inform practice change across these six thematic areas.

## Methods

### Search Strategy

We conducted a structured literature search in PubMed to identify high-quality evidence relevant to perioperative cardiac anesthesiology published between January 1, 2025, and December 31, 2025 using search strategies as previously described.^1^ To ensure capture of high-impact, practice-informing publications, we also performed targeted manual searches of selected general and specialty journals, including *Anesthesiology*, *British Journal of Anaesthesia*, *Anesthesia & Analgesia*, *Journal of Cardiothoracic and Vascular Anesthesia*, *Circulation*, *JAMA Network Open*, *JAMA Surgery*, and *The New England Journal of Medicine*.

Controlled vocabulary terms (e.g., MeSH) and free-text keywords were used to capture the breadth of contemporary perioperative cardiac anesthesia research. Search concepts included (1) the clinical domain (cardiac anesthesia/cardiac surgery), (2) study designs most likely to inform practice (randomized trials, systematic reviews/meta-analyses, guidelines/consensus statements, and registry-based studies), and (3) key perioperative themes (hemodynamic monitoring, echocardiography and ultrasound, enhanced recovery, blood management, analgesia, and organ protection). An example search structure was:

• (“cardiac anesthesia” OR “cardiac anaesthesia” OR “cardiac anesthesiology” OR “cardiac surgical anesthesia”) AND
• (“randomized controlled trial” OR “meta-analysis” OR “systematic review” OR “guideline” OR “consensus” OR “registry”) AND
• (“hemodynamic monitoring” OR “transesophageal echocardiography” OR “TEE” OR “point-of-care ultrasound” OR “POCUS” OR “enhanced recovery” OR “ERAS” OR “blood management” OR “anticoagulation” OR “regional anesthesia” OR “analgesia” OR “renal protection” OR “neuroprotection”).
• Subsequent terms utilized included Cardiac Anesthesiology outcomes, and Cardiac Surgery, and Cardiothoracic Surgery.

### Eligibility Criteria

Search filters were limited to English-language, human studies published in 2025 within the defined time window. We prioritized peer-reviewed publications from high-impact and highly cited journals (typically top-quartile specialty journals and leading general medical journals). We excluded single-patient case reports, editorials, letters, animal studies, and publications from non–peer-reviewed sources.

### Screening and Study Selection

Titles and abstracts were screened independently by three reviewers for relevance to perioperative cardiac anesthesiology and potential to inform clinical practice. Full texts were reviewed when eligibility was unclear or when an article appeared likely to be practice-informing. Discrepancies were resolved by consensus.

During selection, studies were evaluated for methodological rigor (e.g., study design, risk of bias considerations, prespecified outcomes, and appropriateness of analysis), clinical relevance (adult cardiac surgical applicability), and potential impact on perioperative management. Studies were then organized into the following thematic domains: (1) acute kidney injury prevention and organ protection, (2) blood conservation and coagulation management, (3) multimodal analgesia and opioid-sparing strategies, (4) ERAS and postoperative recovery (including ventilation and delirium prevention), (5) point-of-care ultrasound and echocardiographic applications, and (6) workforce sustainability. Initial search results included 1939 studies, which was narrowed down to 639 articles. This was then further screened to generate 24 publications selected for synthesis based on evidence quality, relevance to adult perioperative cardiac anesthesia, and likelihood of influencing practice. Table 1 contains key findings from the highest impact articles out of this group.

**Table 1.** Selected Highest Impact Studies in Cardiac Anesthesiology (2025-2026): Kidney Disease: Improving Global Outcomes (KDIGO), Acute Kidney Injury (AKI), Chronic Kidney Disease (CKD), Cardiopulmonary Bypass (CPB), Prothrombin Complex Concentrate (PCC), Fresh Frozen Plasma (FFP), Acute Normovolemic Hemodilution (ANH); Red Blood Cell (RBC); Non-invasive Ventilation (NIV); Pulmonary Artery Catheter (PAC)

Topic	Authors	Sample size	Conclusions
Biomarker-guided KDIGO bundle (BigpAK-2)	Zarbock et al^4^	N = 1180	A biomarker-triggered KDIGO bundle significantly reduced moderate to severe AKI compared with usual care, supporting structured early AKI prevention.
Nitric oxide conditioning in CKD (DEFENDER)	Kamenshchikov et al^6^	N = 136	Perioperative inhaled nitric oxide reduced AKI and improved 6-month renal function in CKD patients undergoing CPB.
Postoperative 20% albumin infusion (ALBICS-AKI)	Shehabi et al^9^	N = 611	Routine hyperoncotic albumin did not prevent AKI and was associated with increased AKI risk after adjustment.
PCC vs FFP for bleeding (FARES-II)	Karkouti et al^10^	N = 538	4-factor PCC achieved superior hemostatic efficacy compared with FFP in cardiac surgery bleeding.
Acute normovolemic hemodilution (ANH) RCT	Monaco et al^17^	N = 2010	ANH did not significantly reduce RBC transfusion or improve major outcomes in cardiac surgery.
ANH in STS registry	Tanaka et al^18^	N = 16 795	Registry data suggest lower transfusion rates with ANH, particularly when ≥650 mL withdrawn.
Benzodiazepine restriction and delirium	Spence et al^25^	N ≈ 18 000	Restrictive benzodiazepine policy did not significantly reduce postoperative delirium.
Perioperative noninvasive ventilation	Goret et al^29^	N = 216	Pre- and postoperative NIV reduced cardiorespiratory failure in high-risk cardiac surgery patients.
Flow-controlled ventilation (FLOWVENTIN HEARTSURG)	Becker et al^27^	N = 140	Flow-controlled ventilation improved physiologic parameters and showed recovery-relevant signals.
Pulmonary artery catheter use and outcomes	Ju et al^31^	N = 61 405	PAC use was associated with lower short- and long-term mortality in a nationwide cohort.
Cardiac ultrasound consensus statement	Soliman Aboumarie et al^32^	Consensus statement	Standardized terminology, training, and governance for cardiac ultrasound in acute care settings.
ASA guideline on regional analgesia	Joshi et al^19^	628 RCTs reviewed	Strong recommendation for fascial plane blocks in open cardiothoracic surgery.
Opioid-sparing anesthesia meta-analysis	Rauseo et al^20^	N = 58 998	Opioid-sparing strategies reduced opioid use and improved recovery metrics.

## Acute Kidney Injury

Acute kidney injury (AKI) remains one of the most consequential complications following cardiac and other high-risk surgery. Several studies in 2025 offered promising approaches to reducing AKI, including a biomarker-guided care bundle and a phenotype-specific pharmacologic approach with perioperative nitric oxide. Other publications studied the impact of artificial intelligence-guided hemodynamic optimization, and the effects of perioperative albumin and amino acid infusions.

### Biomarker-Guided Kidney Disease: Improving Global Outcomes (KDIGO) Bundle Execution

A preventive care strategy to reduce moderate or severe acute kidney injury after major surgery (BigpAK-2) was a multicenter randomized trial evaluating a KDIGO^3^-based preventive strategy in surgical patients at high AKI risk identified by clinical factors (age ≥75 years; ongoing postoperative vasopressor requirement or need for mechanical ventilation; preexisting stage 3 chronic kidney disease [CKD]; intraoperative exposure to radiocontrast agents) and tubular stress biomarkers (tissue inhibitor of metalloproteinase-2 and insulin-like growth factor–binding protein 7.^4^ A total of 1180 patients (with nearly half of them undergoing cardiac surgery) were randomized, with 1176 included in the primary endpoint analysis. The elements of the preventive care included advanced hemodynamics monitoring (including pulse contour analysis, pulmonary artery catheterization, and transthoracic or transesophageal echocardiography), optimization of volume status, avoidance of nephrotoxins and discontinuation of renin–angiotensin–aldosterone system inhibitors. The primary endpoint was moderate to severe AKI (KDIGO stage 2–3) within 72 hours; this occurred in 84 (14.4%) intervention patients vs 131 (22.3%) controls (OR 0.57; 95% CI 0.40-0.79; *P* = 0.0002; NNT 12 [7-33]). This effect size is clinically meaningful because it targets stage 2–3 AKI rather than the milder creatinine elevations observed in stage 1. However, downstream kidney composite outcomes were not improved; for example, major adverse kidney events to day 90 were similar (61 [11.0%] vs 60 [10.6%]; OR 1.03; CI 0.70-1.50). This study reframes perioperative AKI prevention by showing that early identification of AKI risk through clinical profiles and biomarkers, along with directed clinical interventions, can lead to improvement in at least short-term outcomes. Here, the outcomes improvement was not based on novel therapeutics but instead on reliable execution of a structured KDIGO-based care bundle. Protocolized implementation of evidence-based preventive measures should be considered as the current foundation of perioperative AKI prevention.

### Hemodynamic Optimization Alone: Hypotension Prediction Index as a Negative Control

The hypotension prediction index (HPI) is an artificial intelligence-based technology that has been used to predict intraoperative hypotension (IOH). Habicher and colleagues reported a single-center, single-blinded randomized pilot trial with 150 patients undergoing lung surgery with single-lung ventilation that were randomized to a HPI–guided care group or standard management group.^5^ Patients in the HPI–guided group were managed using a predefined, mechanism-directed algorithm triggered when the HPI reached ≥70%. Preload responsiveness (stroke volume variation (SVV) ≥12%) led to administration of colloid bolus; fluid was continued only if stroke volume increased by ≥10%. If the patient was not fluid responsive or remained unstable in conjunction with a low cardiac index (CI), a dobutamine infusion was started. If the CI was adequate yet MAP remained ≤65 mmHg, vasopressor therapy was initiated. This approach significantly reduced hypotensive burden, with fewer IOH hypotensive episodes and shorter cumulative duration of MAP <65 mmHg. Despite this physiologic improvement, postoperative AKI was not reduced (6.7% [5/75] vs 4.2% [3/71]; *P* = 0.72), nor were postoperative tubular stress biomarkers (tissue inhibitor of metalloproteinase-2 and insulin-like growth factor–binding protein 7) different between groups. The reduction of IOH using predictive mechanism-guided hemodynamic optimization did not reduce AKI in this surgical population, suggesting that meaningful renal protection likely requires coupling hemodynamic stability with additional biologic or phenotype-targeted strategies.

### Nitric Oxide Reduces AKI in Cardiac Surgery Patients With Chronic Kidney Disease (DEFENDER Trial)

Chronic kidney disease (CKD) is characterized by reduced nitric oxide (NO) bioavailability, a deficit that is exacerbated during cardiopulmonary bypass (CPB) by hemolysis and free hemoglobin–mediated NO scavenging. DEFENDER was a randomized controlled trial (RCT) in CKD patients undergoing CPB (136 total; 68 in NO group and 68 in the control group).^6^ Patients in the NO group received inhaled NO at 80 ppm during surgery and for 6 h postoperatively. The incidence of AKI within 7 days was significantly lower with the NO group: 16/68 (23.5%) vs 27/68 (39.7%) (RR 0.59; 95% CI 0.35-0.99; *P* = 0.043), corresponding to an absolute risk reduction of 16.2% (NNT ≈ 6). Importantly, renal function at 6 months favored the NO group (median eGFR 50 [45-54] vs 45 [41-51] mL·min^−1^·1.73 m^−2^; *P* = 0.038). While this is single-center and requires replication, the findings are consistent with NO depletion physiology and support a pharmacologic mechanism-based approach in delivering therapy to phenotype-appropriate patients.

### Amino Acid (AA) Infusion Reduces AKI in Temporary Mechanical Circulatory Support (tMCS): Subgroup Analysis of the PROTECTION Study

Patients receiving tMCS (e.g., intra-aortic balloon pump [IABP], extracorporeal membrane oxygenation [ECMO], and catheter-based percutaneous left ventricular assist device [pLVAD]) experience profound renal stress driven by low cardiac output, ischemia–reperfusion injury, hemolysis, microthrombi formation, and cytokine-mediated inflammation. In the 2024 PROTECTION trial, patients undergoing elective cardiac surgery with CPB were randomized to receive a perioperative intravenous amino acid infusion vs placebo—study findings suggested that amino acids reduced the occurrence of AKI.^7^ Belletti and colleagues have now conducted a subgroup analysis of 232 PROTECTION patients requiring tMCS (80% IABP, 13% ECMO, 9% pLVAD).^8^ In this cohort, AKI incidence exceeded 60% in patients supported with (IABP) and approached 90% in those receiving extracorporeal membrane oxygenation (ECMO). Patients in the intervention arm (n = 112) received intravenous AA at a dose of 2 g/kg of ideal body weight, capped at 100 g per day, for up to 72 hours postoperatively. AKI occurred less frequently in the AA group compared with placebo (44.6% vs 60.8%; relative risk 0.73; 95% confidence interval 0.57-0.94; *P* = 0.01), corresponding to a NNT ≈ 6. After subgroup analysis, this reduction in AKI only remained statistically significant in the IABP group, whereas ECMO and pLVAD subgroups were underpowered and did not demonstrate a statistically significant benefit.

### Routine Postoperative Hyperoncotic Albumin Infusion (ALBICS-AKI Trial)

The ALBICS-AKI trial was a multicenter, open-label randomized study evaluating the ability of a postoperative continuous infusion of hyperoncotic albumin to prevent AKI after high-risk cardiac surgery with CPB.^9^ Patients with preexisting renal dysfunction undergoing on-pump cardiac surgery were included, in addition to those undergoing a combined cardiac surgical procedure or major aortic surgery. 611 patients were evaluated in the primary analysis (307 with albumin; 304 without albumin). Within 6 hours after ICU admission, the intervention group received a 15-hour infusion of 20% albumin; the control group received usual clinician-directed care.^5^ The primary endpoint was creatinine-defined KDIGO stage 1–3 AKI within the first postoperative week. AKI occurred more often in the albumin group: 48.9% (150/307) vs 43.4% (132/304) in usual care. The unadjusted comparison did not reach statistical significance (RR 1.13; 95% CI 0.95-1.34; *P* = 0.18), but after prespecified adjustment for site and baseline eGFR, albumin was associated with a significant increase in AKI risk (adjusted RR 1.12; 95% CI 1.04-1.21; *P* = 0.003). The effect was more pronounced in patients with baseline renal impairment, in whom AKI occurred in 66.2% vs 57.6% (adjusted RR 1.14; 95% CI 1.07-1.22; *P* < 0.001). There were no meaningful differences in major adverse kidney events at 28 days, need for dialysis, ICU or hospital length of stay, or vasopressor-free days. Proposed mechanisms for potential renal injury included an increase in intraglomerular oncotic pressure reducing effective filtration and potential albuminuria-driven tubular inflammation and fibrosis.

## Blood Conservation and Coagulation Management

The need for blood product transfusion remains a central challenge in cardiac surgery; blood conservation strategies and the treatment of coagulopathy directly influence patient outcomes. Several studies in 2025 further investigate the clinical outcomes achieved when prothrombin complex concentrate (PCC) vs fresh frozen plasma (FFP) is administered during the treatment of coagulopathy. Additional studies examine the evolving role of ANH as a core component of blood conservation strategies.

### PCC vs FFP—Efficacy and Thrombin Generation

The FARES-II trial was a multicenter (12 sites in North America), unblinded, randomized noninferiority study enrolling 538 patients who developed clotting factor deficiency-related bleeding following cardiac surgery.^10^ Of these, 420 patients were included in the primary analysis (213 4-factor PCC, 207 FFP). The primary outcome was effective hemostatic response defined as no additional hemostatic interventions required up to 24 hours following initial administration of 4-factor PCC or FFP. The PCC treatment arm demonstrated superior hemostatic efficacy: 78% of PCC patients met hemostatic criteria vs 60% in the FFP group (*P* < 0.001). Furthermore, PCC recipients required fewer second doses (9% vs 19%), fewer platelet transfusions (15% vs 30%) no off-protocol PCC rescue (0% vs 14%; *P* = 0.02).

One of the postulated mechanisms of coagulopathy following CPB is a deficiency in thrombin generation (TG).^11^ Welsby and colleagues conducted a laboratory-based secondary analysis^12^ of a RCT^13^ comparing 4-factor PCC and FFP in 99 patients with persistent post-CPB microvascular bleeding after protamine reversal. The endpoint for transfusion was resolution of bleeding in the surgical field. The primary outcome was change in TG laboratory assays (endogenous thrombin potential [ETP] and peak thrombin generation) through postoperative day 5. Compared to the FFP group, PCC demonstrated significantly greater INR correction (FFP 1.37 vs PCC 1.26; *P* < 0.001), greater ETP (749 vs 1187 nM minutes; *P* = 0.004) and higher peak thrombin (98 vs 124 nM; *P* = 0.033). PCC also increased levels of factor II, VII, IX, X and Proteins C and S compared to FFP. However, by the first postoperative day, TG parameters were similar between groups. These data suggest that PCC promotes TG more rapidly than FFP and hint at mechanisms that support the clinical observations in FARES-II.

### Perioperative Use of Factor Concentrates and Blood Products—A Survey

In a 2019 survey conducted through the American Society of Anesthesiologists (ASA),^14^ FFP was used by more than half of respondents for vitamin K antagonist (VKA) reversal, major operative bleeding, and reversal of direct oral anticoagulants (DOAC) in emergency surgery (the topic of fibrinogen replacement was not evaluated at that time.) To evaluate contemporary practice, Levy and colleagues conducted a 2023 survey^15^ of 27 000 ASA members (1030 respondents, 4% response rate; most respondents practiced in the United States and were evenly distributed between academic and non-academic environments.)

For VKA reversal, PCC was now the preferred therapy in both emergency surgery (63%) and major bleeding (72%). For DOAC reversal, PCC was selected by 77% for emergency surgery and 82% for major bleeding. Access to PCC was variable, with 53% of respondents reporting the ability to use PCC at their own discretion, while 38% reported either needing prior approval or having no access to PCCs. Regarding fibrinogen replacement in major bleeding, 66% of respondents preferred cryoprecipitate, 28% FFP and 6% fibrinogen concentrates.

### Acute Normovolemic Hemodilution

ANH involves collection of autologous blood prior to CPB and reinfusion of this blood volume after separation from CPB. Proposed benefits of ANH include preservation of clotting factors and platelets, improved rheology and reduced need for allogeneic transfusion.^16^

Monaco and colleagues conducted a prospective multinational (11 countries, 32 centers) trial in which 2010 patients undergoing cardiac surgery with CPB were randomized (960 ANH, 955 usual care).^17^ The median volume of withdrawn in the ANH group was 650 mL with an interquartile range of 650–700 mL. The primary endpoint was transfusion of at least one unit of allogeneic red blood cells (RBCs) during hospitalization. RBC transfusion occurred in 27% of the ANH group and 29% in usual care (RR 0.93, CI 0.81-1.07; *P* = 0.34). Secondary outcomes, including 30-day mortality, bleeding complications, ischemic events, chest drain output, and acute kidney injury were also similar.

In contrast to these findings, Tanaka and colleagues evaluated the use and effectiveness of ANH in a retrospective propensity-score matched cohort study using data from the Society of Thoracic Surgeons (STS) Adult Cardiac Surgery Database.^18^ Of 16 795 patients undergoing coronary artery bypass grafting (CABG) and/or valve surgery with CPB between 2020-2023, ANH was reported in 2463 cases (15%). The primary outcome was intraoperative or postoperative transfusion of any blood product. Overall transfusion rates were 31% in the ANH group compared with 36% in the control group, and ANH was associated with 27% lower odds of any transfusion (odds ratio, 0.73; 95% CI, 0.60-0.89). A significant volume-dependent effect was also observed when ANH volume was ≥650 mL: high volume ANH was associated with a 54% reduction in overall transfusion odds (odds ratio 0.46; 95% CI, 0.36-0.59). Cost savings were estimated to be greater than $4 million per 10 000 patients. These two studies further expand the evidence on ANH, but the debate over its meaningful reduction in allogeneic transfusions remains unresolved. The evidence suggesting that maximal benefit is conferred with high volume ANH is persuasive.

## Multimodal Analgesia and Opioid-Sparing Strategies

Cardiothoracic operations are associated with significant postoperative pain and historically managed with high-dose opioids. This approach contributes to adverse effects and delays patient recovery. Multimodal opioid-sparing techniques into perioperative care hold great potential for improving recovery. Recent guidelines, randomized trials, meta-analyses and observational data provide insight into the evolving role of regional anesthesia techniques and multimodal analgesia in cardiac surgery.

### American Society of Anesthesiologists Practice Guideline on Perioperative Pain Management for Cardiothoracic Surgery

Fascial plane blocks are increasingly used in cardiothoracic surgery, but a lack of standardized recommendations contributes to the considerable variability in clinical practice across institutions. Joshi and colleagues provided evidence-based recommendations for perioperative pain management using regional anesthesia specifically tailored for cardiothoracic and other surgical specialties. The task force reviewed 628 randomized clinical trials, of which 124 were included in pooled analyses.^19^

For open cardiothoracic surgeries (e.g., CABG, valve surgery, and lobectomy), pooled analysis of five RCTs (n = 535) demonstrated that erector spinae plane (ESP) and serratus anterior plane (SAP) blocks reduced 24-hours pain scores beyond the minimal clinically important difference (MCID) compared to control patients (i.e., no intervention, placebo, or sham.) Eight trials (n = 730) also showed reduced opioid consumption, with a mean decrease of 60 oral morphine equivalents (MME). Based on moderate-strength evidence, use of fascial plane blocks in open sternotomy and thoracotomy was strongly recommended. In minimally invasive procedures (20 trials; n = 1611), single-injection fascial plane blocks reduced 24-hour pain but did not exceed the MCID threshold. Therefore, only a conditional recommendation was issued due to low-quality evidence and modest clinical benefit.

The authors emphasized that their ability to make stronger recommendations was limited by inconsistent outcome reporting, poor standardization of block nomenclature, small sample sizes, and variable methodological quality. Nonetheless, fascial plane blocks should be strongly considered for integration into the routine postoperative care of patients undergoing open cardiothoracic operations.

### Opioid-Sparing Anesthesia

Rauseo and colleagues conducted a systematic review and meta-analysis of 27 studies (8 RCTs, 19 observational studies; n = 58 998) comparing opioid-sparing strategies against traditional opioid-based paradigms in adult cardiac surgical patients.^20^ Primary outcomes included total opioid consumption (MME), ICU length of stay, duration of mechanical ventilation, and postoperative pain scores.

Opioid-sparing techniques reduced cumulative perioperative opioid consumption (−2.48 MME; *P* < 0.001), shortened ICU length of stay (OR 1.32), reduced duration of mechanical ventilation (OR 1.46; 95% CI 1.24-1.72; *P* < 0.001), and improved pain scores at 12 hours postoperatively (OR 1.18; 95% CI 1.07-1.30; *P* = 0.002). Secondary outcomes that included reduced postoperative nausea and vomiting, lower persistent opioid use, earlier mobilization, and improved patient satisfaction favored opioid-sparing approaches, though inconsistent reporting precluded pooled analysis. These findings support the routine use of perioperative opioid-sparing techniques to improve recovery metrics, but the magnitude of the clinical benefit warrants further study.

### Parasternal Intercostal Blocks in CABG

Truncal blocks targeting the anterior branches of the intercostal nerves have emerged as a technique to reduce postoperative opioid use in cardiac surgical patients. Korkmaz Toker et al conducted a prospective, randomized, single-blind trial (n = 75) comparing superficial parasternal intercostal plane block (SPIPB - by anesthesiologist using ultrasound after sternal closure), deep parasternal intercostal plane block (DPIPB—by surgeon under direct vision intraoperatively), and standard care (chest drain site infiltration) in elective CABG patients.^21^ Both SPIPB and DPIPB significantly reduced tramadol consumption compared to controls (141 mg and 95 mg vs 176 mg, respectively; *P* < 0.001) and better dynamic pain control at 24 hours (median pain score 6 and 5 vs 8 in control; *P* < 0.001) with DPIPB being most effective. Both block groups were associated with shorter median extubation times (7.0 (6.0–8.0) vs 8.0 (7.0–9.0) *P* = 0.006 hours for control).

### Rectus Sheath Block Added to Parasternal Block in Cardiac Surgery

Parasternal blocks reduce the pain of sternotomy but inconsistently cover the sensory dermatomes innervating epigastric chest drain sites. This superiority, single-blind, prospective RCT by Strumia et al involved 58 patients undergoing cardiac surgery via median sternotomy to evaluate whether adding bilateral rectus sheath blocks (RSB) to parasternal blocks improves analgesia and respiratory function during the first 24 postoperative hours.^22^ At extubation, maximum pain scores were significantly lower in the RSB group (median 4 (4-4) vs 5 (4-5); *P* = 0.03). The addition of an RSB significantly reduced median postoperative morphine consumption by 2 mg over 24 hours (95% CI, 0.0 to 2.0; *P* < 0.01). And peak expiratory flow was superior at 6, 12, and 24 hours. Postoperative nausea and vomiting were also significantly reduced (7% vs 35%; 95% CI 0.05-0.77; *P* < 0.01).

### Peripheral Nerve Blocks for Enhanced Recovery After Cardiac Surgery

Sharkey et al conducted a retrospective observational study of 413 cardiac surgical patients within an established ERAS program.^23^ A total of 170 patients received peripheral nerve blocks (PNB; pectoro-intercostal fascial block and/or rectus sheath block). Primary outcomes included cumulative opioid consumption, ICU length of stay (LOS), postoperative mobility, and incidence of postoperative atrial fibrillation (POAF). The authors found that patients receiving PNBs achieved earlier ambulation (15 vs 18 hours; *P* = 0.037), had a shorter ICU LOS (44 vs 50 hours; *P* = 0.024), and lower rates of POAF (27% vs 33%; *P* = 0.045). Cumulative opioid consumption was not significantly different between the PNB and control groups. It is intriguing to consider that the benefits of postoperative PNB could be exerted via mechanisms beyond simple analgesia (potentially involving respiratory mechanics and modulation of sympathetic tone). The study findings are limited by the small sample size and the single-center, retrospective and observational study design.

## Postoperative Care in Cardiothoracic Surgery

Individuals requiring cardiac surgery are commonly burdened by multiple comorbidities which would prolong their recovery from inherently stressful procedures. Enhanced recovery after cardiac surgery is an evolving multidisciplinary effort to provide the smoothest, fastest recuperation possible. Recent and ongoing research is diverse and examines nearly all aspects of care in search of opportunities for improvement. A selection of impactful studies furthering our understanding of cardiac perioperative medicine follows, where aspects of medication choice, respiratory protection, invasive monitoring, and comprehensive care bundles are examined.

### Benzodiazepine-Free Cardiac Anesthesia and Postoperative Delirium

Benzodiazepines are commonly employed perioperatively for cardiac surgical patients for anxiolysis, sedation, limitation of sympathetic surge, and prevention of awareness under anesthesia but may also increase the likelihood of postoperative delirium (POD) in a vulnerable population. The magnitude of both the beneficial and detrimental effects of these medications have been called into question recently.^24^

Spence and colleagues studied benzodiazepines and the rate of delirium after cardiac surgery in a pragmatic cluster-randomized crossover trial at 20 institutions in North America.^25^ Hospitals were randomized to a restricted or liberal policy, defined as at least 0.03 mg/kg predicted body weight midazolam equivalents intraoperatively, in 8 week blocks for up to 18 crossover periods with few other restrictions. Over 9000 patients were assigned to each group with excellent protocol adherence. The liberal and restricted groups had a 14.9% and 14.0% incidence of POD (OR 0.92 (CI 0.84 to 1.01), respectively, which was not statistically significant. In the secondary analyses, patients undergoing surgery during restrictive periods had fewer episodes of POD and per-protocol patients had a statistically significant 1.1% reduction in delirium incidence if benzodiazepines were avoided. Excluding patients who received benzodiazepines within 24 hours of surgery from the restrictive group also generated statistical significance. No patient-reported increase in intraoperative awareness was found. Overall, this was a large, pragmatic, and rigorous trial that found no clinically significant increase in POD with the use of benzodiazepines, which the authors believe is a multifactorial syndrome for which medication choice is but a small inciting component.

### Individualized Flow-controlled vs Pressure-controlled Ventilation in Cardiac Surgery (FLOWVENTIN HEARTSURG Study)

Beyond typical ventilatory parameters monitored to prevent ventilator-induced lung injury there has been increasing attention paid to mechanical power imparted into the respiratory system. Flow control ventilation is a novel ventilator mode that minimizes power by controlling gas flow rates during inspiration and expiration.^26^ Becker and colleagues studied whether flow control ventilation in the perioperative cardiac surgical population could reduce lung injury.^27^ In their study FLOWVENTIN HEARTSURG, they randomized 140 cardiac surgical patients at a single center to flow control-continuous mandatory ventilation (FCV) or pressure control-continuous mandatory ventilation (PCV) with the aim of reducing lung injury as measured by the inflammatory marker IL-8. Secondary outcomes included incidences of postoperative pulmonary and extrapulmonary complications and hospital LOS. Initial low tidal volumes and individualized PEEP titration were standardized in both groups.

At several time points after surgery there was a significantly lower IL-8 plasma concentration in the FCV group but not at the predefined timepoint 6 hours after cardiopulmonary bypass. Multiple ventilatory parameters were improved in the FCV group including mechanical power, compliance, and minute ventilation for normocapnia despite an average lower PEEP, larger driving pressure, and larger tidal volume, though the different methods of measuring pressure confound these comparisons. PaO_2_/FiO_2_ ratios were similar intraoperatively but significantly better in the FCV group upon reaching the ICU as were postoperative ventilator weaning time and duration of mechanical ventilation. Pulmonary complications such as hypoxemia and reintubation were similar between groups overall, although the FCV group was found to have more frequent mild hypoxemia but less frequent severe hypoxemia. The composite extrapulmonary complication rate (including acute kidney injury, delirium, and tamponade) was higher in the control group (OR 2.65:^:^ 95% CI 1.11 to 6.37) but no individual complication was significantly different in incidence. This single-center study has some chance imbalance between the two patient groups, and no correction for the multiple comparisons was undertaken. Despite these limitations, mechanical power minimization shows promise for protecting the lungs of vulnerable patients.

### Perioperative Noninvasive Ventilation and Postoperative Morbidity

Noninvasive positive pressure ventilation treats many of the cardiorespiratory diseases which are common in the cardiac surgical population such as heart failure and chronic obstructive pulmonary disease, in addition to postoperative issues such as pulmonary derecruitment and edema.^28^

Goret and colleagues initiated a single-center randomized trial in which 216 high-risk adult patients undergoing planned cardiac surgery were assigned to standard care with or without twice-daily noninvasive ventilation (BIPAP with face mask) for 4 days before and after surgery.^29^ The primary outcome was the cumulative incidence of a composite of cardiorespiratory failure at one month with secondary outcomes of the composite outcome at 3 months, the components of the primary outcome, and LOS in the ICU and the hospital. Despite poor postoperative protocol adherence, the intervention group had a 24% absolute reduction of the composite outcome, from 79.8% to 55.1% (RR 0.69; 95% CI 0.57-0.84; *P* = 0.0001). The incidence of acute respiratory failure improved from 45% to 16.8% (RR 0.37; 95% CI 0.23-0.60; *P* < 0.0001). The composite outcome (non-NIV 79.8% vs NIV 56.1%; RR 0.70; 95% CI 0.58-0.85; *P* = 0.002) at 3 months was similar. The novel intervention of pre- and post-surgical noninvasive ventilation had a strong effect on postoperative respiratory outcomes and may benefit high-risk patients.

### Impact of Pulmonary Artery Catheter Use on Mortality After Cardiac Surgery

The use of pulmonary artery catheterization in cardiac surgical patients offers the prospect of continuous, detailed quantification of hemodynamic parameters when it is most needed; data to support this use however is lacking. Randomized trials outside of cardiac surgery have found little benefit, though one recent observational trial found some benefit in cardiogenic shock. Multiple retrospective observational studies in cardiac surgery have found variable associations but largely either no benefit or a signal for harm.^30^ Utilizing a Korean health insurance database, Ju and colleagues retrospectively analyzed a 61 405 patient cohort for the incidence of 1-year all-cause mortality for those undergoing surgery with and without pulmonary artery catheter use^31^ They found a reduction in 1-year mortality with PAC use (OR 0.81; 95% CI 0.76-0.87; *P* < 0.001) and a reduction in 30-day mortality (OR 0.76; 95% CI 0.69-0.83; *P* < 0.001). Patients undergoing CABG benefited the most, especially off-pump procedures. Subgroup analysis found that only low-volume centers benefited from the use of a pulmonary artery catheter with no difference in high volume centers.

## Cardiac Ultrasound and Point-Of-Care Ultrasound (PoCUS)

Cardiac ultrasound is undergoing a fundamental transformation from a comprehensive, specialty-based imaging modality into a real-time diagnostic and decision-making platform guiding immediate clinical management. This evolution directly impacts the practice of cardiothoracic anesthesia and critical care, where perioperative physiology and hemodynamic management are intertwined. In 2025, four publications were identified which help solidify this transition into the perioperative and critical care spaces.

### Cardiac Ultrasound Consensus Statement

In 2025, a multi-society consensus statement was published to serve as a practical guide for the utilization of cardiac ultrasound (CUS) in various acute care settings and for clinicians from various disciplines.^32^ It establishes CUS as an indispensable tool in guiding rapid, real-time clinical decision-making in unstable patients during cardiovascular emergencies. Emphasis is placed on a systematic and pathophysiological approach to diagnosis and management. CUS should be a first-line tool in the evaluation of undifferentiated shock or unexplained hemodynamic instability, but that reliance on isolated echocardiographic measurements (e.g., EF, TAPSE, or IVC size) can lead to misinterpretation or delayed treatment. Clinicians performing CUS are encouraged to employ a systematic approach to integrate findings with an understanding of cardiovascular physiology and to prioritize serial assessments instead of drawing conclusions from a single exam. The utility of CUS is further bolstered when integrating lung and organ-specific ultrasound into a comprehensive assessment of hemodynamics.

The statement introduces a standardized, tiered framework that matches various clinical applications with levels of operator training and system capabilities. It defines specific terminologies associated with ultrasound in cardiovascular emergencies and critical care including emergency echocardiography, focused cardiac ultrasound (FoCUS), point-of-care ultrasound (PoCUS), targeted or goal-directed echocardiography, critical care echocardiography, and focused transesophageal echocardiography (fTOE). The authors attempt to clarify scope of practice, governance, and competency-based training requirements, including the establishment of two levels of competence for practitioners in emergency and ICU environments: the independent basic operator and the advanced operator. Overall, the statement advocates a multimodal, systems-based approach, supported by standardized training, quality assurance, and continuing medical education to ensure that CUS is applied safely and effectively across various acute care specialties.

### Intraoperative FoCUS: Feasibility for Resident Education

FoCUS is now a core component of anesthesiology medical education.^33^ A barrier to integration into resident education is the relative lack of intraoperative scanning which is commonly felt to be impractical in operating room workflows. The authors assess the feasibility of teaching FoCUS to anesthesiology residents in the intraoperative environment.^34^ 160 exams (performed by 4 senior residents) as part of an FoCUS curriculum were retrospectively analyzed. They found that residents successfully performed intraoperative FoCUS exams with image acquisition rates comparable to experienced anesthesiologists. Parasternal views were the most easily obtained; lower abdominal and extremity operations were associated with highest success rates. Trendelenburg positioning also improved imaging.

## Cardiothoracic Anesthesiology Workforce

The contemporary physician workforce continues to experience unprecedented strain with staffing shortages, demographic pressures, shifting generational expectations, ethnogeographic health disparities, and systemic hurdles in training and retention.^35^ In 2025, several articles provide a snapshot of the contemporary cardiothoracic anesthesiology workforce and address topics ranging from the unique challenges faced by underrepresented minority (URM) cardiac anesthesiologists, characteristics of the global cardiac anesthesia workforce, and the preparedness of cardiac anesthesiologists in dealing with low-performing colleagues.

### A Call to Diversity

Despite years of initiatives designed to increase diversity in medicine, underrepresented minorities (URMs) remain disproportionately represented in anesthesiology. Modest gains have been made in medical school admissions and residency recruitment, but these advances have not translated into equivalent levels of faculty representation. Persistent disparities in promotion, compensation, retention, and leadership roles suggest that recruitment per se is insufficient to achieve equity within the specialty.

Sumler and colleagues argue that URM faculty have a different experience in the academic anesthesiology environment that stems from fundamental inequities in the domains of professional advancement and compensation.^37^ Effective mentoring, networking and coaching programs require recognition, willingness and intentionality on the part of anesthesiology department leaders to prioritize these faculty development endeavors in the face of clinical productivity pressure. There is also growing recognition in professional anesthesiology societies that pathways into national leadership roles need to be developed; the creation and efforts of the Society of Cardiovascular Anesthesiologists Diversity, Equity and Inclusion Committee is a notable example of tangible progress in this domain but there is still much progress to be made.

### Global Workforce Survey

Mittnacht and colleagues conducted a cross-sectional multilingual web-based survey to create a snapshot of the global cardiac anesthesiology workforce based on responses from over 3400 cardiac anesthesiologists across 99 countries.^36^ A central finding was the considerable variability in formal cardiac anesthesia and transesophageal echocardiography (TEE) training. Nearly half of respondents reported completion of an accredited fellowship, but almost 45% of respondents lacked formal TEE training and relied instead on workplace-based practical training. Significant regional differences in practice patterns (e.g., who performs intraoperative TEE, manages CPB, and staffs cardiac intensive care units) were also identified.

The survey also highlighted factors impacting workforce well-being. Burnout was common (31.7% of respondents) and independently associated factors included compensation, clinical work hours, age, call-related factors, work-life balance, and geographical region. A major limitation to this survey was that the primary mechanism of study distribution was via cardiac anesthesia society membership lists—this may have created selection bias. Nonetheless, an intriguing picture of the global cardiac anesthesia practice is presented.

### Low-Performance in Cardiothoracic and Vascular Anesthesia

Samara and colleagues present an international cross-sectional web-based survey (465 responses spanning 57 countries) with the primary objective of defining low performance in cardiothoracic and vascular anesthesia (CTVA).^37^ Secondary outcomes included assessment of institutional approaches to identify, report, and deal with low performance.

Eighteen survey statements defining low-performing CTVA anesthesiologists (LPC-CTVA) were developed based on published literature and expert discussion. The characteristics assessed spanned the domains of clinical competence, knowledge, communication/ethics and compliance with standards. Out of these 18 items, survey respondents achieved consensus (defined as ≥70% agreement by respondents) on 13 statements. Only 11.7% of those surveyed reported a structured institutional system for managing low-performing colleagues, and less than half of respondents felt confident to intervene. These findings help create a consensus-based definition of LPC-CTVA and support the need for the development of clearer policies and structured training for the institutional management of these individuals.

## Conclusions

Notable cardiothoracic anesthesiology publications of 2025 covered a wide breadth of topics ranging from (ANH) and cardiac anesthesiology workforce diversity. There was incremental advancement in the prevention of AKI, and our growing understanding of the use of PCC compared to FFP was corroborated by shifts in clinical paradigms for the treatment of coagulopathy. Consensus statements were released regarding PoCUS in response to the growing need for standardization in both practice and simple nomenclature. The potential for truncal and peripheral nerve blocks to play an increasingly prominent role in improving patient recovery after cardiac surgery was demonstrated. The impact on postoperative outcomes through various other interventions, including the use of pulmonary artery catheters and noninvasive positive pressure ventilation was also evaluated. Overall, these findings reflect a specialty that is focused on practical, patient-centered improvement and continue to build on the foundation laid by our predecessors.
